# Zika Induces Human Placental Damage and Inflammation

**DOI:** 10.3389/fimmu.2020.02146

**Published:** 2020-09-01

**Authors:** Kíssila Rabelo, Luiz José de Souza, Natália Gedeão Salomão, Lara Nascentes Machado, Priscila Gomes Pereira, Elyzabeth Avvad Portari, Rodrigo Basílio-de-Oliveira, Flávia Barreto dos Santos, Laura Dias Neves, Luciana Faes Morgade, David William Provance, Luiza Mendonça Higa, Amilcar Tanuri, Jorge José de Carvalho, Marciano Viana Paes

**Affiliations:** ^1^Laboratório de Ultraestrutura e Biologia Tecidual, Universidade do Estado do Rio de Janeiro, Rio de Janeiro, Brazil; ^2^Faculdade de Medicina de Campos, Rio de Janeiro, Brazil; ^3^Laboratório Interdisciplinar de Pesquisas Médicas, Instituto Oswaldo Cruz, Fiocruz, Rio de Janeiro, Brazil; ^4^Anatomia Patológica, Instituto Fernandes Figueira, Rio de Janeiro, Brazil; ^5^Anatomia Patológica, Universidade Federal do Estado do Rio de Janeiro, Rio de Janeiro, Brazil; ^6^Laboratório de Imunologia Viral, Instituto Oswaldo Cruz, Fiocruz, Rio de Janeiro, Brazil; ^7^Hospital Geral Dr. Beda, CEPLIN – Uti Neonatal Nicola Albano, Rio de Janeiro, Brazil; ^8^Centro de Desenvolvimento Tecnológico em Saúde, Fiocruz, Rio de Janeiro, Brazil; ^9^Laboratório de Virologia Molecular, Universidade Federal do Rio de Janeiro, Rio de Janeiro, Brazil

**Keywords:** immune response, histopathology, ultrastructure, cytokines, flavivirus

## Abstract

In Brazil, an epidemic of Zika virus (ZIKV) infections was declared in 2015 that coincided with alarming reports of microcephaly in newborns associated with mother infection. Although the virus has placental tropism, changes in the tissue morphology and immunity of infected patients have not yet been elucidated. Here, we investigated the histopathological and ultrastructural changes along with the immunological profile and the BDNF expression in rare placental material. Tissues were obtained in the 2015–2016 Brazilian epidemic, of ten ZIKV-infected patients during pregnancy, five resulting in cases of fetal microcephaly and five non-microcephaly, compared to five non-infected control placentae. Viral antigens were only detected in samples from the ZIKV infected patients. Infected placentae presented histopathological severe damage, while the ultrastructural evaluation showed abnormal organelles, such as clusters of virus-like particles consistent with the ZIKV dimensions. Increased infiltration of CD68^+^ and TCD8^+^ cells, expression of MMPs, cytokines (IFN-γ and TNF-α) and other immunological mediators (RANTES/CCL5 and VEGFR-2) confirmed excessive inflammation and vascular permeability dysfunction. An evaluation of BDNF showed a decrease that could modulate neuronal damage in the developing fetus. The placental changes caused by ZIKV are not pathognomonic, however, the data provide evidence that this infection leads to severe placental injury.

## Graphical Abstract

Schematic drawing of placental cells detected infected with ZIKV and cytokines and mediators produced by the mononuclear inflammatory infiltrate. The scheme represents the functional unit of the placenta (chorionic villus) and the maternal region (decidua), comprised of cytotrophoblast (CTB), endothelial cells (En), syncytiotrophoblast (STB), mesenchymal cells (MS), fibroblasts (Fi), Hofbauer (Hf), macrophages (MØ), decidual cells (DC), and extracellular matrix, containing collagen (COL). In the placentae of infected patients, ZIKV antigens were detected in all these cells and there was an increase in the number of macrophages and Hofbauer cells, which are responsible for the production of matrix metalloproteinases that degrade the collagen as well as TNF-α that activates and attracts other immune cells. There was also an increase in T CD8^+^ lymphocytes, responsible for the production of IFN-γ, which further activates macrophages. In endothelial cells, there was large expression of VEGFR-2 and RANTES, which increases vascular permeability and are able to induce macrophages migration.

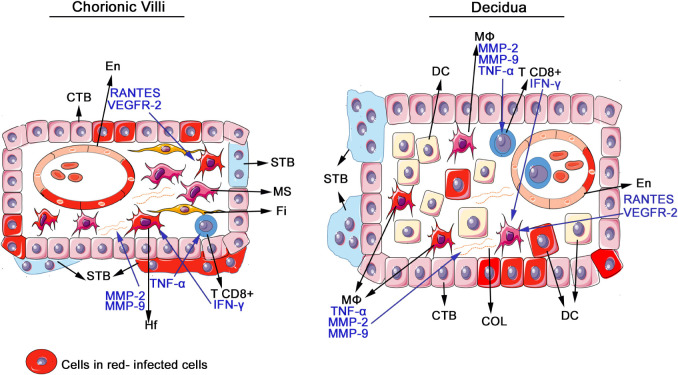

## Introduction

Zika virus fever has emerged as an important arbovirus disease whose transmission has impacted numerous regions worldwide. Its etiological agent, Zika virus (ZIKV), was first isolated more than seventy years ago in the Zika forest of Uganda from the blood of sentinel Rhesus monkeys during a 1947 study on yellow fever transmission. For nearly 60 years, serological evidence of ZIKV infections in humans was only detected sporadically on the continents of Africa and Asia. This rare occurrence changed in 2007 when an epidemic appeared with a large incidence of the disease in Micronesia that was followed by another in Polynesia in 2013 before its subsequent appearance worldwide (1). In Brazil, a Zika epidemic was declared in 2015 from its appearance in the Northeast of the country that rapidly propagated across the country (1, 2). This epidemic was soon followed by alarming reports of microcephaly in fetuses and newborns that were associated with mothers infected by ZIKV, which led to a declaration by the World Health Organization (WHO) of a public health emergency of international concern (3–5). In 2017, the Brazilian Ministry of Health adopted new parameters to measure the cephalic perimeter and identify cases of microcephaly, following the WHO recommendation. For boys, the measurement is equal to or less than 32.5 cm and for girls, 31.5 cm (6).

Many authors have reported on the capacity of ZIKV to infect neurons and other neuronal cells that most likely detrimentally affect their function and contribute to congenital Zika syndrome (CZS), which has as its main characteristics microcephaly and ventriculomegaly (1, 3–5, 7, 8). In this scenario, studies aiming to understand the mother-fetus interface of ZIKV vertical transmission have been strongly recommended (9). In the vertical transmission, one major barrier is the placenta, a highly specialized organ that ensures the fetus’ development, by allowing the exchange of nutrients, solutes and acting as physiological barrier against toxic molecules and pathogens, such as viruses (9, 10). Estimates are that the viral genome can be detected in the placenta of 20–50% of pregnant women exposed to ZIKV (11). However, the mechanism by which ZIKV crosses the placenta to establish an infection in a fetus has not been completely elucidated.

To date, ZIKV has been identified in amniotic fluid and a range of placental cells (syncytiotrophoblasts, cytotrophoblasts, decidual, and endothelial cells) as well as cells of the maternal immune system present in the placenta, such as macrophages and dendritic cells (4, 12–15). At present, definitive evidence is lacking for the histopathological changes associated with a ZIKV infection during an active immune response in the placenta of pregnant patients. Defining these changes could have major implications in understanding the impact of a positive ZIKV diagnosis for a pregnant mother on the severity of the condition for their fetus as a predictor for microcephaly.

Here, we present the clinical aspects of 10 pregnant patients infected with ZIKV during the outbreak that occurred in Rio de Janeiro between 2015 and 2016. Five of these pregnancies ended with the birth of infants that presented with microcephaly (ZIKV^+^MIC^+^) and the other five with infant that did not present with microcephaly (ZIKV^+^MIC^–^). Microcephaly was the only clinical aspect of the newborn considered as it is detectable at the time of delivery and is one of the most prominant characteristics of CZS. Here, we describe the histopathological features observed in both groups of infected placentae with a comparison to five, uninfected control placentae. In addition, we report on the detection of viral antigens in placental cells, some of immune cells, cytokines, proinflammatory mediators, ultrastructural changes and the detection of virus-like particles by electron microscopy. Finally, we evaluated the expression of brain derived neurotrophic factor (BDNF), an essential factor for fetal brain development, which may be one of the determinant proteins that contribute to the severity of microcephaly due to vertical transmission in ZIKV infection.

## Materials and Methods

### Ethics Statements and Sample Collection

All procedures performed during this study were approved by the Ethics Committee of the Oswaldo Cruz Foundation/FIOCRUZ (CAEE: 65924217.4.0000.5248) and by the Ethics Committee of Faculty of Campos Medicine/Benedito Pereira Nunes Foundation (CAEE: 65924217.4.3001.5244). Consent and permission were obtained from patients and participating institutions. Ten placentae were collected from women infected by ZIKV during pregnancy that resulted in the birth of five babies with birth microcephaly (ZIKV^+^MIC^+^) and five with normal cranial circumference at birth (ZIKV^+^MIC^–^). After delivery, placenta samples were fixed in 10% formalin or 2.5% glutaraldehyde. Samples from ZIKV infected women were collected at the Hospital Plantadores de Cana, Hospital Geral Dr. Beda from Campos dos Goytacazes, Rio de Janeiro, Brazil and Hospital de Clínicas Padre Miguel, from Rio de Janeiro, Brazil. As a reference control, five samples of term placenta from healthy donors were included. All samples were collected between 2015 and 2016 that coincide with the ZIKV epidemic in Brazil.

### Histopathological Investigation

Fixed placenta samples were dehydrated in ethanol, clarified in xylene and blocked in paraffin. Tissue sections (4 μm thick) were mounted onto glass sides, deparaffinized in three baths of xylene and rehydrated with decreasing concentrations of ethanol (100 to 70%) before staining with hematoxylin and eosin for histological examination. Prepared specimens were observed by light microscopy (Olympus, Japan) and digital images captured using Image-Pro Plus software version 7. All images were coded to blind evaluators to ZIKV^±^ and MIC^±^ prior to analysis.

### Morphometry

Collagen was revealed by Picro Sirius Red and slides were observed under polarized light microscopy (Olympus). Fifty fields were randomly acquired at 400x magnification from across the placenta samples (Zika-infected and control) and the area of collagen was measured to calculate the percentage of collagen area (collagen area/total area of the image).

### Immunohistochemistry Assays

Paraffin-embedded tissue sections (4 μm) were mounted onto glass slides, deparaffinized in xylene and rehydrated with alcohol. Antigen retrieval was performed by heating the tissue in the presence of citrate buffer by 20 min at 60°C (pH 6.0) (Spring Bioscience, Pleasanton, CA, United States). Next, tissues were blocked for endogenous peroxidase with 3% hydrogen peroxidase in methanol and rinsed in PBS (pH 7.4) (Spring Bioscience). Sections were incubated in Protein Blocker solution (Spring Bioscience) for 5 min at room temperature to reduce non-specific binding. Samples were then incubated overnight at 4°C with anti-human monoclonal antibodies against: flavivirus E protein (4G2 – produced in house, diluted 1:200), CD8 [C8/144B] (DAKO Cytomation, United States, diluted 1:200), CD68 [KP1] (Biocare Medical, United States, diluted 1:100), CD4 [SP35] (Cell Marque, United States, diluted 1:100), RANTES/CCL5 [F11] (Santa Cruz Biotechnology, United States, diluted 1:100), TNF-α [KT31] (Abbiotec, United States, diluted 1:200), IFN-γ [P01579] (Abbiotec, diluted 1:200), VEGFR-2 [E3712] (Spring Bioscience, diluted 1:50), Zika NS1 [SQab1609] (Arigo, United States, diluted 1:200) or BDNF [SAB2108004] (Sigma-Aldrich, United States, diluted 1:50). After three washes, sections were incubated with an anti-mouse or anti-rabbit IgG-HRP conjugate (Spring Bioscience) for 40 min at room temperature. HRP was revealed by its activity on the chromogen substrate diaminobenzidine (Dako, United States) and sections were counterstained in Mayer’s hematoxylin (Dako). For negative controls, samples were incubated with either only primary antibodies or secondary HRP conjugated antibody prior to exposure to chromogen substrate.

### Quantification of Positive Cells by Immunohistochemistry

Slides were observed on an Olympus BX 53F microscope. For each specific antibody stain, images from 50 random fields were acquired at 1000x magnification using the software Image Pro version 7 from samples originating from all placentae (ZIKV infected and controls). The number of positive cells were quantified in each of the 50 fields and after segregating the fields to the three conditions (ZIKV^+^MIC^+^; ZIKV^+^MIC^–^, and ZIKV^–^MIC^–^) the mean number of positive cells per field was calculated. All image acquisitions were performed by an individual blinded to the diagnosis associated with the tissue sample. Figures present representative fields to best convey the quantification results.

### *In situ* Hybridization

*In situ* hybridization studies were performed on placentae tissue sections from all cases and controls using a commercial RNA scope Target Probe (catalog #463781; Advanced Cell Diagnostics, United States) that was complementary to sequences 1550–2456 of the ZIKV genome. Pretreatment, hybridization and detection techniques were performed according to manufacturer’s protocols. The probe-target complex was revealed by alkaline phosphatase activity on the chromogen substrate nitroblue tetrazolium and bromo-chloro-indolyl phosphate.

### Immunofluorescence Assay

Paraffin-embedded tissue sections (4 μm) were mounted onto glass slides, deparaffinized in xylene, exposed to decreasing concentrations of ethanol from 100 to 70% and then fully rehydrated in PBS with decreasing alcohol content to 0%. Next, slides were incubated in PBS with 1% bovine serum albumin for 30 min and then permeabilized 30 min in PBS with 0.5% Triton X-100 at room temperature. After washing, slides were co-stained overnight at 4°C with a 1:200 dilution of a mouse IgG monoclonal anti-Zika NS1 [SAB2108004] (Arigo) and a rabbit IgG monoclonal anti-human CD163 [EPR19518] (Abcam, United Kingdom). After washing, sections were incubated with an Alexa 488-conjugated rabbit anti-mouse IgG and Alexa 555-conjugated goat anti-rabbit IgG, diluted 1:200. After washing and mounting, slides were imaged using a Zeiss LSM 510 Meta confocal microscope (Carl Zeiss, Germany).

### Molecular Diagnosis by RT-PCR

Human serum samples collected on the day of delivery were obtained from six patients and sourced for the isolation of viral RNA using Qiagen RNAEasy. RNA was quantified with the Qubit RNA HS Assay Kit (Thermo Fisher Scientific, United States) and purity was evaluated using NanoDrop ND-1000 Spectrophotometer (NanoDrop Technologies, United States) followed by the synthesis of cDNA using First-Strand Synthesis System^®^ (Invitrogen, United States). The amplification reaction was routinely performed by combining the reverse transcription of viral RNA and the subsequent Taq polymerase amplification in a single reaction. The TaqMan PCR Master Mix kit (Invitrogen) was used to amplify the oligonucleotide set utilized targeted the intergenic region of the Membrane/Envelope as described by Lanciotti, 2008 (16). Results were conclusive in two samples.

### Molecular Diagnosis by PRNT_90__%_

A plaque-reduction neutralization test (PRNT) was performed to detect the presence of neutralizing antibodies against ZIKV in the serum obtained from the six patients mentioned above. Serum samples were incubated at 58°C for 30 min and then subjected to a series of two-fold dilution beginning from 1:5 to 1:2,560 that were individually incubated with an equal volume containing 100 plaque forming units (PFU) of ZIKV (strain MR 766) at 37°C. After 1 h, the virus-plasma mixture was inoculated onto a confluent monolayer of VERO cells. After an additional hour, inoculum was removed and a semisolid medium (1.4% carboxymethylcellulose in alpha-MEM supplemented with 1% fetal bovine serum) was layered on top of the cells, which were cultured for 5 days before fixation with 4% formaldehyde. Cells were stained with a crystal violet dye solution and the PRNT end-point titers were expressed as the reciprocal of the last serum dilution showing a ≥90% reduction in plaque counts. A PRNT_90_ titer ≥20 was considered positive for the presence of neutralizing antibodies against to ZIKV.

### Electron Microscopy Analysis

Placental tissue samples were fixed with 2.5% glutaraldehyde in sodium cacodylate buffer (0.1 M, pH 7.2), post-fixed with 1% buffered osmium tetroxide, dehydrated in an acetone series (30, 50, 70, 90, and 100%) and embedded in EPON that was polymerized at 60°C for 3 days. Ultrathin sections (60 nm) were contrasted with uranyl acetate and lead citrate before visualization on a JEOL 1001 transmission electron microscope (Jeol Ltd., Tokyo, Japan).

### Statistical Analysis

Data were analyzed with GraphPad Prism software v 6.0 (GraphPad Software, San Diego, CA, United States) using non-parametric statistical tests. Significant differences between the analyzed groups were determined using the One-Way ANOVA test with *post hoc* Tukey, with a threshold of *P* < 0.05.

## Results

### Clinical Data of Pregnant Women (ZIKV^+^) With Babies That Did Not Present Microcephaly (MIC^–^)

*Case 1*: A 42 year-old patient that reported exanthema and pruritus in the first trimester of gestation. Her serology for IgG against cytomegalovirus, rubella, dengue, toxoplasmosis and HIV were negative. At 39 weeks of gestation, she delivered a baby boy by cesarean that presented a cephalic circumference of 35 cm. The placenta weighed 640 g.

*Case 2*: A 23 year old patient that reported fever, arthralgia, exanthema and pruritus in the third trimester of gestation. Her IgM serology was positive for Zika, the PRNT_90__%_ was positive (Supplementary Table S1) and the qPCR was positive for Zika in serum (820 copies/ml) and urine (160 copies/ml). Her IgG serology was positive for cytomegalovirus and rubella. The test for dengue NS1 was negative. At 38 weeks of gestation, her baby girl was born by cesarean delivery that presented with cephalic circumference of 37 cm. The placenta weighed 555 g.

*Case 3*: A 21 year old patient that reported fever in the second trimester of pregnancy. Her IgM serology for ZIKV was positive and non-reactive for dengue, chikungunya, rubella, toxoplasmosis, HIV, syphilis and cytomegalovirus. The PRNT_90__%_ was positive for neutralizing antibodies. Her IgG serology was positive for cytomegalovirus and rubella. At 41 weeks of gestation, her baby girl was born by cesarean delivery that presented a cephalic circumference of 36 cm.

*Case 4:* A 26 year old patient that reported exanthema and arthralgia in the second trimester of gestation. The qPCR was positive for Zika in serum (690 copies/ml) and the PRNT_90__%_ was positive for neutralizing antibodies. At 37 weeks of gestation, her baby boy was born by cesarean delivery that presented a cephalic circumference of 36.5 cm.

*Case 5:* A 34 year old patient that reported exanthema and pruritus in the third trimester of gestation. At 38 weeks of gestation, her baby girl was born by cesarean that presented a cephalic circumference of 34 cm.

### Clinical Data of Pregnant Women (ZIKV^+^) With Babies That Presented Microcephaly (MIC^+^)

*Case 6:* A 29 year old patient that reported exanthema and pruritus in the second trimester of gestation. Her IgM serology was positive for Zika and the PRNT_90__%_ was positive for neutralizing antibodies. At 38 weeks of gestation, her baby girl was born by cesarean that presented a cephalic circumference of 30 cm.

*Case 7:* A 24 year old patient that reported exanthema and pruritus in the second trimester of gestation. Her IgM serology for dengue, herpes, chikungunya, rubella, toxoplasmosis, HIV and cytomegalovirus were non-reactive. Her IgG serology was positive for dengue, herpes, rubella and cytomegalovirus, and negative for toxoplasmosis. A dengue NS1 test was negative. At 38 weeks of gestation, her baby boy was born by cesarean that presented a cephalic circumference of 29 cm. The newborn also had ventriculomegaly.

*Case 8:* A 35 year old patient that reported exanthema, shiver and pruritus in the first trimester of gestation. Her IgM serology was positive for Zika and the PRNT_90__%_ was negative for neutralizing antibodies. Her IgG serology for cytomegalovirus and rubella were positive while negative for dengue, toxoplasmosis and HIV negative. At 38 weeks of gestation, her baby girl was born by cesarean with a cephalic circumference of 29 cm.

*Case 9*: A 25 year old patient that reported exanthema and pruritus in the third trimester of gestation. Her IgM serology was positive for Zika and the PRNT_90__%_ was positive for neutralizing antibodies. At 37 weeks of gestation, her baby girl was born by cesarean with a cephalic circumference of 27 cm. The placenta weighed 565 g.

*Case 10:* A 28 year old patient who experienced fever in the third trimester of pregnancy. Her IgM serology for dengue, chikungunya, rubella, toxoplasmosis, HIV, syphilis and cytomegalovirus were non-reactive, and positive for ZIKV. Her IgG serology was positive for cytomegalovirus and rubella. At 38 weeks of gestation, her baby boy was born by cesarean delivery with a cephalic circumference of 28 cm. The baby also presented arthrogryposis, with lower and upper limb involvement. The placenta weighed 670 g.

### Histopathological Analysis

**FIGURE 1:**
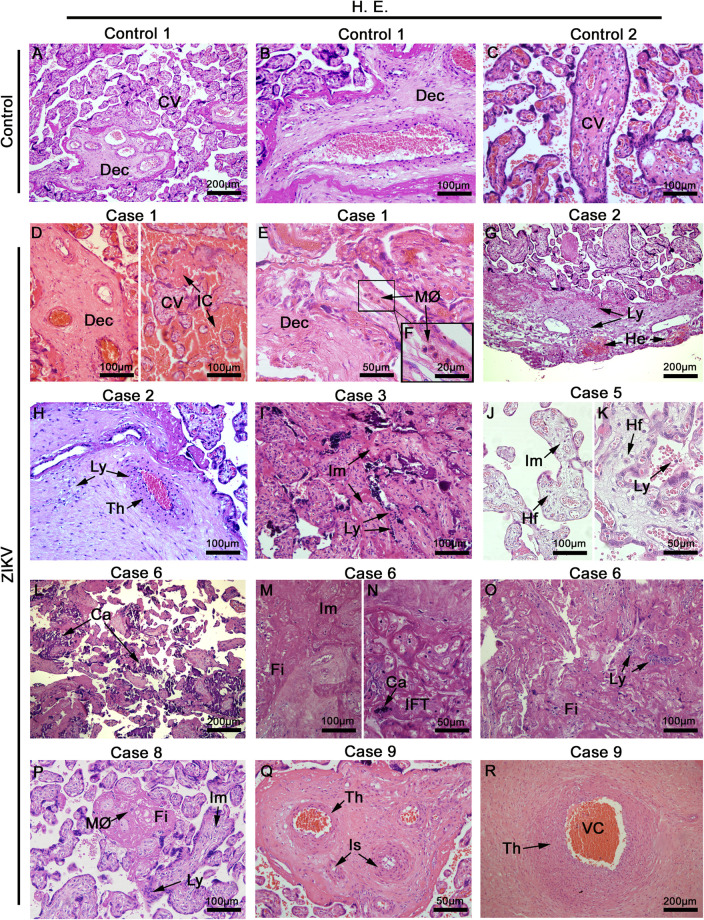
Placentae histopathology. **(A–C)** Placentae from non-ZIKV patients stained with H&E and presenting normal features: maternal decidua (Dec) and chorionic villi (CV). **(D–R)** Placentae from ZIKV infected patients that presented a range of different alterations such as VC, vascular congestion; IC, intervillous congestion; MØ, macrophage infiltrate; Ly, lymphocytic infiltrate villous; He, hemorrhage; Th, endothelial thickening; Im, immature chorionic villi; Hf, Hofbauer cells; Ca, calcification; Fi, fibrin areas; IFT, infarct, and Is, ischemia.

The histopathological analysis of control samples showed a regular arrangement of the decidual layer and normal chorionic villi that included syncytiotrophoblasts, cytotrophoblasts and endothelial cells (Figures 1A–C), which suggested that the collection and fixation of placental samples was adequate. For the evaluation of the placenta samples from ZIKV mothers, the full range of samples were imaged and qualified for histological alterations. In infected placentae, we observed relevant damage in the decidua and chorionic villi (Figures 1D–R). Large areas of immature chorionic villi were evident that included inflammatory changes seen as acute deciduitis and villositis, chronic villositis (lymphocytic infiltrate), fibrous endothelial thickening, vascular and intervillous congestion and focus of intervillositis. Other alterations were present: calcification, edema, fibrin deposits and villous hypoplasia. In addition, a few incidences of ischemic lesions were identified as infarct and decidual vasculopathy (fibrinoid necrosis) (Supplementary Table S1). No correlations were apparent with the presentation of microencephaly in infants after birth suggesting that the changes observed represented the effects that can be expected in placenta from a maternal ZIKV infection that are not predictive for the impact on fetus development.

### Ultrastructural Alterations and Zika Virus Particles

**FIGURE 2:**
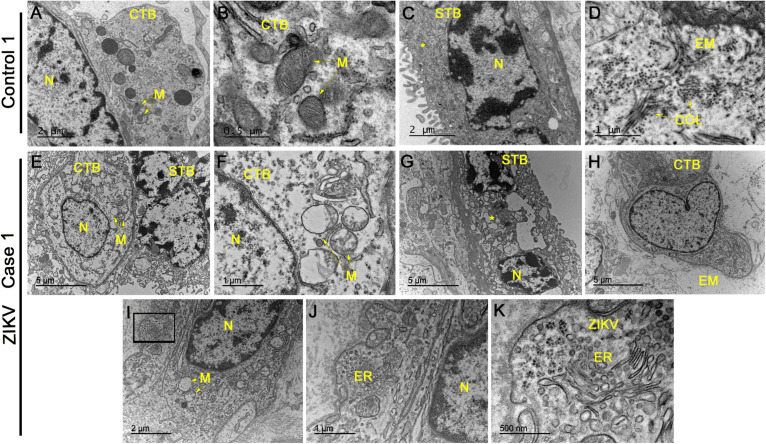
Electron microscopy analysis of placental sections showed alterations and virus-like particles in ZIKV infected samples. **(A–D)** Electron microscopy images of ultrathin sections of placental tissue from a single, non-ZIKV infected mother that exhibited regular cytotrophoblasts (CTB), syncytiotrophoblasts (STB), nucleus (N), mitochondria (M), and collagen filament structures (COL). **(E–H)** Electron micrographs of ultrathin sections of placental tissue from different ZIKV-infected mothers showing CTB with alterations in the cytoplasm, nuclear variation (N) and swollen mitochondria with a loss of cristae and membrane rupture. The identified STB presented an enlargement of vesicles and apoptotic bodies (asterisks) along with an absence of normal membrane extensions and evidence of microvesicle secretion. The extracellular matrix (EM) did not present collagen filaments. **(I–K)** Identification of clusters of virus-like particles that were positioned near the endoplasmic reticulum (ER) of CTB and ∼25 nm in diameter, which is consistent with the dimensions of ZIKV.

No evidence of ultrastructural changes was observed in placental cells from control patients, represented in images form a single control 1 (Figures 2A–D), which again suggests the collection and treatment of samples maintained their structural integrity. The cytotrophoblasts presented normal aspects for all organelles, including the mitochondria and the nucleus, which was heterochromatic (Figures 2A,B). Syncytiotrophoblasts presented an electron dense cytoplasm, as expected, with heterochromatic nuclei (Figure 2C). In the extracellular matrix, collagen filament structures were readily identified in transverse and longitudinal sections (Figure 2D). In contrast, the analysis of infected placentae showed cytotrophoblasts with little nuclear variation and an electron lucid cytoplasm containing swollen mitochondria showing a loss of cristae and ruptured membranes (Figures 2E,F). The syncytiotrophoblasts aspects were extensively modified with an enlargement of vesicles and apoptotic bodies along with an absence of their normal membrane extensions and secretions of microvesicles (Figures 2E,G). An investigation of the extracellular matrix across the images of placenta samples from the different donors did not reveal collagen filaments, which suggests a decrease in this matrix component (Figure 2H). Multiple occurrences of clusters were identified that presented a profile reminiscent of virus-like particles, which were often positioned adjacent to the endoplasmic reticulum of cytotrophoblast cells. These particles were measured to have a diameter of ∼25 nm in diameter, which is consistent with the dimensions of ZIKV (Figures 2I–K).

### Viral Detection in the Placentae

**FIGURE 3:**
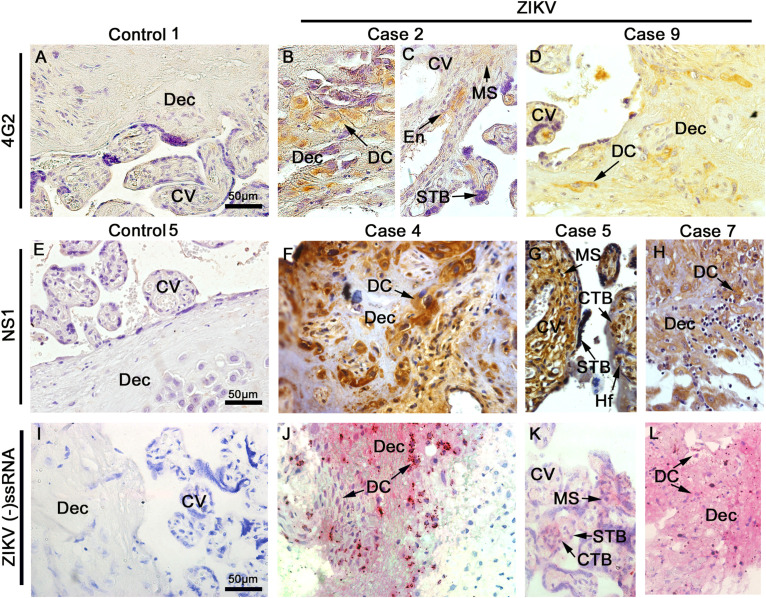
Detection of viral antigens. Tissue sections were probed by immunohistochemistry for the E **(A–D)** and NS1 **(E–H)** antigens of ZIKV as well as by *in situ* hybridization for the genome of ZIKV **(I–L)**. Representative images of control placentae **(A,E,I)** showing the absence of reactivity for the antigens and genome of ZIKV. Representative images of placenta from ZIKV^+^ placenta, independent of the cephalic circumference of the infant, show: **(B–D)** the presence of E protein in decidual cells (DC) of decidua (Dec), mesenchymal cells (MS), endothelial cells (En), and syncytiotrophoblast (STB) of chorionic villi (CV) that was independent of the cephalic circumference of the infant; **(F–H)** NS1 protein detected in decidual cells (DC) of decidua (Dec), mesenchymal cells (MS), Hofbauer (Hf), syncytiotrophoblast (STB) and cytotrophoblast (CTB) of chorionic villi (CV); and **(J–L)** Detection of ZIKV RNA negative strand by *in situ* hybridization in decidual cells (DC) of decidua (Dec), mesenchymal cells (MS), syncytiotrophoblast (STB) and cytotrophoblast (CTB) of chorionic villi (CV).

The placental tissue samples were screened for the presence of ZIKV E and NS1 protein using immunohistochemistry. These viral antigens were detected in all samples obtained from infected patients, while immunostaining was negative in samples of control placentae (Figures 3A,E). The E structural protein was detected in decidual cells and in syncytiotrophoblasts as well as endothelial and mesenchymal cells of chorionic villi (Figures 3B–D). The NS1 protein was also detected in cytotrophoblasts, syncytiotrophoblasts and mesenchymal cells, moreover in Hofbauer cells of chorionic villi and in decidual cells (Figures 3F–H). Viral antigens were detected mainly within the cytoplasmic region of cells with minor to indefinite staining in the nuclear area. The anti-NS1 antibody used in these assays is ZIKV specific, therefore, it was able to differentiate ZIKV from other flaviviruses. Additionally, the replication was also confirmed by *in situ* hybridization using a probe that anneals only to the negative strand of the ZIKV RNA, which revealed the presence of this RNA in decidual cells, syncytiotrophoblasts, cytotrophoblasts and villous mesenchymal cells (Figures 3J–L). All controls were negative for the immunohistochemistry and *in situ* hybridization (Figure 3I). The staining pattern observed strongly suggests that the replication of ZIKV was in progress at the time of birth.

### Characterization of Cell Subpopulations

**FIGURE 4:**
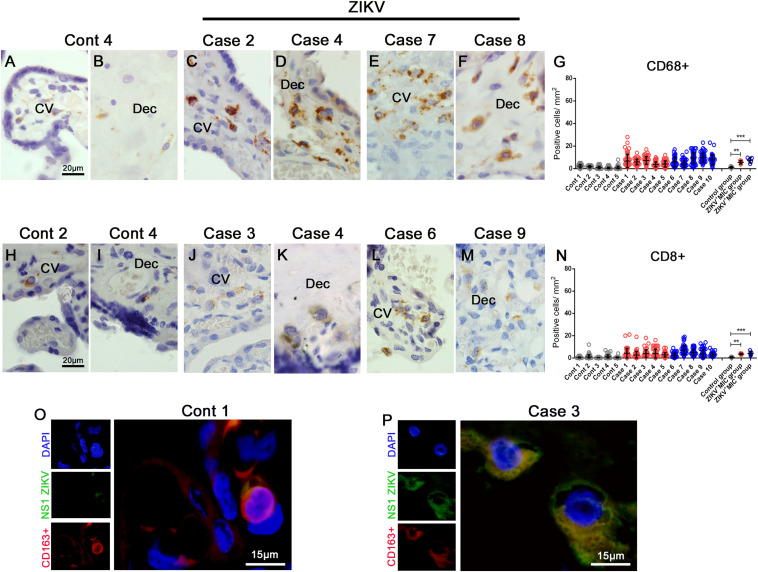
Increased cellularity of mononuclear cell subpopulations in ZIKV-infected placental tissues. CD68 and CD8 were detected in placenta samples by immunohistochemistry. **(A–B)** CD68^+^ cells in decidua and chorionic villi of control placenta. **(C–F)** CD68^+^ cells in decidua and chorionic villi of ZIKV infected placentae. **(G)** Quantification of CD68^+^ cells showing a 5 or 6-fold increase in placentae of ZIKV^+^MIC^–^ and ZIKV^+^MIC^+^ groups, respectively. **(H,I)** CD8^+^ cells in decidua and chorionic villi of control placentae. **(J–M)** CD8^+^ cells in decidua and chorionic villi of ZIKV infected placentae. **(N)** Quantification of CD8^+^ cells showing a 7 or 8-fold increase in the tissues of ZIKV^+^MIC^–^ and ZIKV^+^MIC^+^ groups, respectively. **(O,P)** Immunofluorescence for the presence of ZIKV NS1 protein (green) and CD163 (red; biomarker for macrophages) showing colocalization. Nuclei were stained using DAPI (blue). **(O)** ZIKV NS1 antigen was not detected in the control placenta. Data are represented as mean ± SDM. Asterisks indicate differences that are statistically significant between groups (***p < 0.001).

To gain further insight into the subpopulations of immune cells that could be migrating to inflamed tissues, immunohistochemistry was performed to characterize the cell types present in the placentae. A significant upsurge in the number of CD68^+^ cells were detected in both groups of infected placentae (ZIKV^+^MIC^–^ and ZIKV^+^MIC^+^), which suggested a recruitment of macrophages and hyperplasia caused by the infection in the basal decidua and Hofbauer cells (Figures 4C–F). The increase of macrophages were quantified to be 5- and 6-fold in placentae of ZIKV^+^MIC^–^ and ZIKV^+^MIC^+^ groups, respectively (Figure 4G). Even though T CD8^+^ cells were found in the same areas (Figures 4J–M), few CD4^+^ cells were detected within the tissues (Supplementary Figure S1). The T CD8^+^ lymphocytes were increased 7- and 8-fold in the tissues from ZIKV^+^MIC^–^ and ZIKV^+^MIC^+^ groups, respectively (Figure 4N). The control placentae showed a low number of positive cells for both markers (Figures 4A,B,H,I). Additional evidence for the replication of ZIKV in macrophages was observed by the colocalization of NS1 protein with the CD163 marker for differentiated macrophages in dual stained immunofluorescent images (Figure 4P). As expected, no signals were observed for NS1 in control tissue (Figure 4O).

### Cytokines and Mediators Profile in the Placentae

**FIGURE 5:**
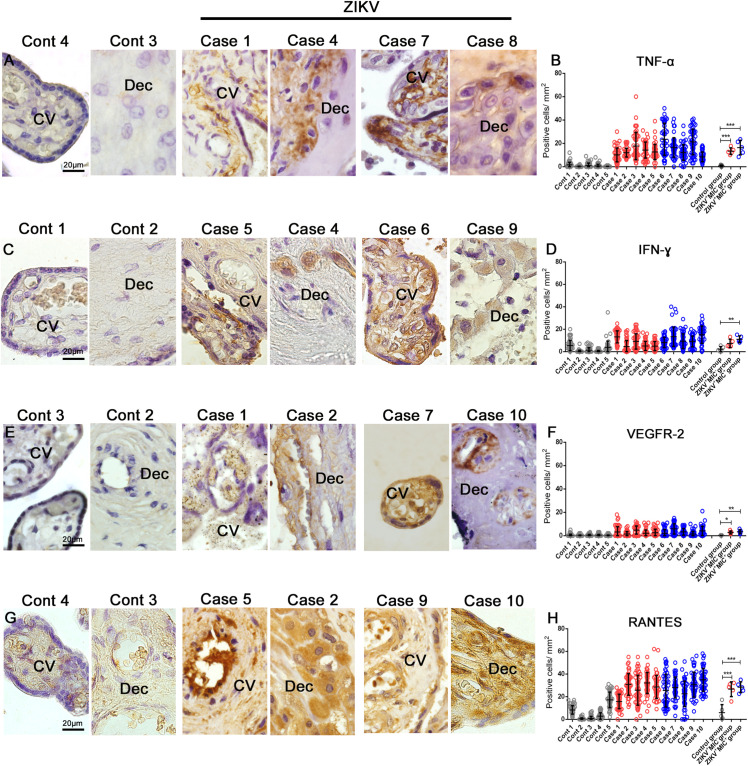
Cytokine-producing cell profile. Detection of TNF-α, IFN-γ, VEGFR-2 and RANTES/CCL5 by immunohistochemistry show **(A)** TNF-α in cells of chorionic villi in control placentae (Left panel) and ZIKV infected placentae (Right panel). **(C)** Production of IFN-γ in macrophages as well as endothelial cells in chorionic villi and decidual cells of the decidua of control placentae (Left panel) and ZIKV infected placentae (Right panel). **(E)** VEGFR-2 was expressed in endothelial cells of decidua and chorionic villi in control placentae (Left panel) and ZIKV infected placentae (Right panel). **(G)** RANTES/CCL5 present mainly in the endothelium and Hofbauer cells located within the chorionic villi and decidual cells and syncytiotrophoblasts of the decidua in control placentae (Left panel) and ZIKV infected placentae (Right panel). Quantification of the cells positive for **(B,D,F,H)** Quantification of the number of cells expressing TNF-α **(B)**, IFN-γ **(D)**, VEGFR2 **(F)** and RANTES/CCL5 **(H)** showed an increased expression of local pro-inflammatory cytokines and mediators in ZIKV positive placentae compared to controls. Data are represented as mean ± SDM. Asterisks indicate differences that are statistically significant between groups (**p < 0.01) or (***p < 0.001).

Based on the inflammatory infiltrate observed in H&E stained sections and the detection of an increase in number of immune cells in infected placentae, the production of cytokines and mediators were investigated. The expression of TNF-α and IFN-γ was evaluated due to their participation in a pro-inflammatory response. In addition, Additionally, the markers VEGFR-2 and RANTES/CCL5 were included as they have been implicated with an alteration in vascular permeability. In control samples, all markers were detected at low levels (Figures 5A–H). TNF-α expression was diffuse in Hofbauer and mesenchymal cells in the chorionic villi and in decidual cells (Figure 5A). Its expression was 12-fold higher in the ZIKV^+^MIC^–^ group and 16-fold higher in the placenta from the ZIKV^+^MIC^+^ group (Figure 5B). IFN-γ was found mostly in decidual cells and decidual macrophages (Figure 5C), with a 3- and 5-fold increase in the ZIKV^+^MIC^–^ and ZIKV^+^MIC^+^ groups, respectively (Figure 5D). The expression of VEGFR-2 was found in endothelial and mesenchymal cells in chorionic villi as well as in circulating macrophages within the vessels and in endothelial cells in decidua (Figure 5E). This receptor had an increased expression level of 11- and 13-fold in the ZIKV^+^MIC^–^ and ZIKV^+^MIC^+^ groups, respectively (Figure 5F). The chemokine RANTES/CCL5 was detected mainly in the endothelium and in Hofbauer cells located within the chorionic villi and in decidual cells and syncytiotrophoblasts of the decidua (Figure 5G). RANTES/CCL5 was expressed 4- and 5-fold upward in ZIKV^+^MIC^–^ and ZIKV^+^MIC^+^ groups, respectively, compared to control group (Figure 5H). The statistical analysis of the expression of all these markers determined that they were significantly increased in the ZIKV^+^ patient placentae compared to ZIKV^–^ control tissues (Figures 5B,D,F,H).

### Changes in Placental Collagen and Matrix Metalloproteinases

**FIGURE 6:**
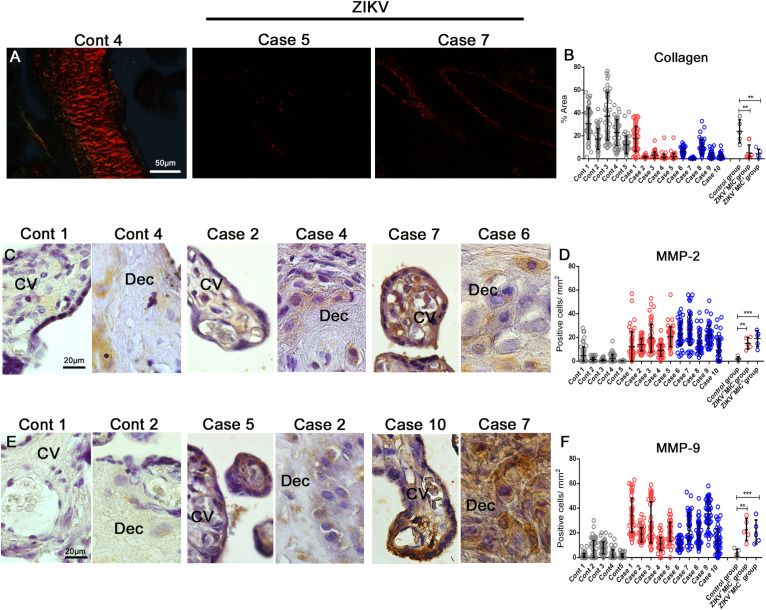
Detection and quantification of collagen, MMP-2 and MMP-9 collagenases expression. **(A)** Collagen detection by Picro Sirius Red staining in placental tissues. **(B)** The percent collagen area was quantified in all cases that showed a decrease in the expression of collagen in infected placentae. **(C–E)** Detection of MMP-2 and MMP-9 in decidual cells and cells located within the chorionic villi in both control and ZIKV infected placentae. **(D–F)** Quantification of the number of cells expressing MMP-2 and MMP-9 showed an increased expression in ZIKV infected placental tissues. Data are represented as mean ± SDM. Asterisks indicate differences that are statistically significant between groups (**p < 0.01) or (***p < 0.001).

The absence of collagen in the electron micrographs was confirmed by its specific staining with Picro Sirius Red, which showed that a ZIKV infection led to a drastic decrease in placental collagen (Figure 6A). The reduction was 5- and 9-fold in the tissues from ZIKV^+^MIC^–^ and ZIKV^+^MIC^+^ groups, respectively (Figure 6B). The levels of collagen can be altered by matrix metalloproteinases (MMPs), which can degrade collagen and are known to play a crucial role in pregnancy. MMPs are increased during inflammation from their production by the infiltrated immune cells. An investigation of MMP-2 and MMP-9 levels showed that both proteins were expressed at low levels in decidual cells and chorionic villi cells of control placenta. However, their expression was substantially elevated in the placenta from ZIKV^+^ mothers and displayed a diffuse pattern (Figures 6C,E). MMP-2 levels were 6- and 8-fold greater in ZIKV^+^MIC^–^ and ZIKV^+^MIC^+^ groups, respectively (Figure 6D). MMP-9 increased 11- and 10-fold in ZIKV^+^MIC^–^ and ZIKV^+^MIC^+^ groups, respectively (Figure 6F).

### BDNF Expression in Placental Cells

**FIGURE 7:**
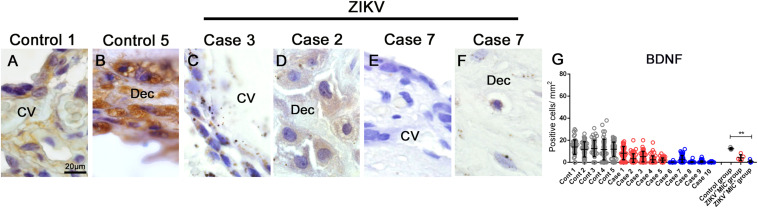
BDNF detection and quantification in placental tissues. **(A–F)** Detection of BDNF in cells of chorionic villi and maternal decidua of controls and ZIKV infected placentae by immunohistochemistry. **(G)** A quantification of the number of cells expressing BDNF showed a decreased in the expression of this hormone in ZIKV^+^ MIC^+^ cases. Data are represented as mean ± SDM. Asterisks indicate differences that are statistically significant between groups (**p < 0.01).

Lastly, the placental expression of an important neurotrophine related to neurogenesis, BDNF, was detected and quantified by immunohistochemistry. In control samples, BDNF was readily detected by an intense and diffuse signal in cells of decidua and chorionic villi (Figures 7A,B). The intensity was noticeably diminished in samples from ZIKV^+^ placentae and the number of BDNF expressing cells was considerably lower (Figures 7C–F). By quantification, there were 12.35 positive cells for BDNF/mm^2^ in the control group and 4.25 positive cells for BDNF/mm^2^ in the ZIKV + MIC group, which had no statistical difference. In the ZIKV^+^MIC^+^ group, only 0.7 BDNF positive cells/mm^2^ were detected, which was significantly different (Figure 7G).

## Discussion

Here, we investigated the impact of a maternal ZIKV infection on placental tissue in patients who gave birth to babies with or without microcephaly during the ZIKV outbreak in Brazil. The histopathology of the ZIKV infection on placenta in Brazilian patients has been studied previously by our group and some alterations are common in most cases, such as a delayed villi maturation, fibrin deposits, calcification and inflammatory changes in villi and the decidual layer (4, 15). The main alteration observed in the placentae of the cases studies was the delay in villi maturation, confirmed by other groups (17–19). These histopathological alterations are similar to those described in the placentae from ZIKV^+^ women in French Guiana, such as villitis, intervillositis, calcification, infarct, ischemia, inflammatory infiltrate and fibrin deposits (20–22). Overall, the placental changes discovered in ZIKV infection are non-pathognomonic and often have particular characteristics in different patients.

In addition to confirming the histopathological modifications associated with a ZIKV infection, we examined cells of the placentae at an ultrastructural level. The intracellular damage caused by the virus was observed principally in mitochondria of cytotrophoblasts. There also was an increase in the vesicles present in the syncytiotrophoblasts and it was difficult to detect collagen filaments in the samples from infected patients. While different ultrastructural damage has been observed previously with ZIKV infections, this is the first report on modifications in the structure of organelles in placental tissue obtained from a human *in vivo* infection (4, 23). In addition to the organellar alterations, the presence of clusters of virus-like particles were identified positioned near to the endoplasmic reticulum of cytotrophoblasts.

The viral antigens E and NS1 as well as the genomic RNA of ZIKV were detected in numerous cells throughout the placentae, in both fetal or maternal portions that included trophoblasts. These results provide further proof that placental cells are susceptible and permissive to ZIKV infection, which is consistent with the hypothesis that ZIKV can reach the developing fetus by progressive cell to cell infections that can penetrate the placental barrier (4, 15, 21, 24–27). It is important to note that results presented here provides ample evidence that the ZIKV established a persistent, replicating infection in the placenta months after the reported onset of the acute infection based on the detection of virus by RT-PCR, electron microscopy and immunohistochemistry as well as markers of replication from *in situ* hybridization and viral antigens at the moment of delivery.

Hofbauer cells and decidual macrophages are residents in the placenta that have a regulatory role in pregnancy to maintain a homeostatic environment, which is essential for fetal development (28). We detected a large increase in the number of macrophages in placenta from infected mothers. In addition, fluorescence microscopy captured colocalization of ZIKV NS1 in CD163^+^ activated macrophages that suggested these cells were sites of virus replication. Macrophages have been previously identified as principal targets for ZIKV infection and could provide a pathway for the vertical transmission of ZIKV through their activation that can lead to a prominent and diffuse hyperplasia (15, 21, 29–31). Infected CD163^+^ cells have already been suggested as one of the factors associated with virus delivery to the fetus that lead to ZIKV-induced fetal damage (32).

In the samples analyzed in this study, we found an expressive increase in the numbers of T CD8^+^ lymphocytes. Regla-Nava and colleagues suggested that the lack of T CD8^+^ cells, which occurs in mice exhausted by a previous infection, such as dengue fever, could facilitate ZIKV infection (33). Most patients in our study were IgG negative or not reactive for dengue suggesting that none were compromised by a previous infection that have facilitated their ZIKV infection. Even the exception, case 7, still had an increase in the migration of T CD8^+^ cells to the placenta. This increase in T CD8^+^ lymphocytes has been observed in non-human primates after the decrease of viremia, which suggests a protective role for T CD8^+^ cells in controlling ZIKV replication (34). In humans, while few reports have shown the cellular profile in the placenta from ZIKV infected mothers, our observation from a pregnancy of only 15 weeks showed the same characteristics (15). Another case report that presented as positive for T CD8^+^ lymphocytes was even more expressive for T CD4^+^ (21), which was unlike our samples where the number T CD4^+^ cells were insufficient for quantification.

The increase in macrophages and T CD8^+^ cells characterizes a chronic inflammatory environment in the placenta, with lesions such as deciduitis and villitis observed in all cases (35). Immunity is essential for the development of a pregnancy, from implantation to delivery, and it is now known that maternal immune activation (MIA) is dynamic and normally very effective at preventing viral infections (36–38). However, ZIKV appears to establish a placental infection that bypasses the MIA and promotes inflammation. This environment can be initiated through the release of pro-inflammatory cytokines, such as TNF-α and IFN-γ whose levels are exacerbated in this study, which induce chemotaxis and cellular activation that also increase the expression of MHC-1 for even more intense actions by cytotoxic lymphocytes. The evaluation of these cytokines was prioritized in our study, since TNF-α is an evident cytokine in an inflammation process, while IFN-γ activity has already been associated with microcephaly in studies with neuronal cells and patients serum (39).

The levels of VEGFR-2 receptor and RANTES/CCL5 mediator were also elevated in the tissues studied, which can lead to an increase in vascular permeability and could cause a large circulatory dysfunction as the fibrin deposits. Fibrin deposits in the placenta can observed in cases of spontaneous abortion, premature birth and fetal death, which suggests a direct affect on the development of the fetus and pregnancy (40). The expression of VEGFR has already been related to other pathologies in the placenta and RANTES/CCL5 has been previously observed in ZIKV placental infection (15, 31, 41, 42). Their changes can lead to edema and a failure in the distribution of nutrients as well as hormones necessary to maintain tissue homeostasis.

In our study, placental tissues infected with ZIKV showed a large decrease in the expression of collagen, which corroborated findings from the ultrastructural analysis and was consistent with the higher production levels of MMP-2 and MMP-9 enzymes. The extracellular matrix (EM) provides an environment conducive to placental development, regulating cellular functions such as signaling, proliferation, migration and invasion. EM is composed of proteoglycans, glycosaminoglycans and has collagen as its main structural component. Most placental collagen is type III (around 60%), followed by type I collagen (approximately 30%) and the other types are IV, V, and VI (43, 44). MMPs play a role in the implantation, vasodilatation and separation of fetal membranes, developing a crucial role in collagen degradation according to the signaling by hormones (45, 46). It is known that the inflammatory environment leads to the release of MMPS by immune cells (47). Due to the highly inflammatory environment caused by ZIKV infection, it is certain that the immune cells have secreted and caused this increase in MMPs and consequently the degradation of collagen, leading to malefic tissue remodeling for placental homeostasis. The increase in MMPs in infected placentas may have contributed to the immaturity of the villi.

In all analyses of immune cells and cytokines profile, there were no statistical differences observed between the samples of ZIKV^+^MIC^–^ and ZIKV^+^MIC^+^ groups. The only exception was the IFN-γ, where there was no difference between the ZIKV^+^MIC^–^ group and the control, even though there was an increase. Our results showed that there is a large inflammation response in the placenta from mothers with a ZIKV infection, but if it has a role in the changes in brain fetal development, it is subtle.

In the absence of a clear role for the inflammation response in the presentation of microcephaly, the amount of placental BDNF, a factor described as a determinant for fetal brain development, was evaluated (48, 49). BDNF is a neurotrophic factor that is produced in placental tissue and plays an important role in cytotrophoblast differentiation and proliferation (50, 51). Additionally, this neurotrofin promotes neuronal growth and differentiation in the central and peripheral nervous system during fetal development (52, 53). The placentae of patients infected with ZIKV, especially from the group that presented infants with microcephaly, showed a decrease in BDNF expression, which suggests that BDNF levels in the placenta could serve as predictive marker for the extent of damage during fetal brain development. However, it is important to emphasize that BDNF would not act alone in the damage to the development of the fetal nervous system, since there was no direct relationship between the amount of BDNF and the size of the head circumference. Other biomarkers must be discovered and related to those already mentioned in the literature for elucidate fetal damage with direct infection to fetal neuronal cells (32, 54).

## Conclusion

As summarized in Graphical abstract, our work corroborates other studies that show that many placental cells are susceptible and permissive to ZIKV infection. In addition, there is a large involvement of immune cells and pro-inflammatory cytokines in the infected tissue, leading to changes in activation and recruitment of circulating cells as well as alterations in the extracellular matrix and vascular permeability. Statistically, the inflammatory response in the placenta did not have a straight impact on the presentation of microcephaly, subtle differences were evident and an expanded study may uncover relevant biomarkers. BDNF, which is important in the development of the brain, was found in the placenta, and could be a promising marker to predicting the impact of a maternal ZIKV infection on fetal brain alterations if considered together with others. As infected, pregnant women are the main target population for a possible vaccine against Zika, knowledge of the immune cells involved in placental inflammation, including the cytokines and mediators released by local cells, in the course of disease is crucial for its development. The discoveries from this study highlight this need and advance the current description placental change that contribute to congenital ZIKV pathogenesis.

## Data Availability Statement

The raw data supporting the conclusions of this article will be made available by the authors, without undue reservation.

## Ethics Statement

The studies involving human participants were reviewed and approved by the Ethics Committee of the Oswaldo Cruz Foundation/FIOCRUZ (CAEE: 65924217.4.0000.5248) and the Faculty of Campos Medicine/Benedito Pereira Nunes Foundation (CAEE: 65924217.4.3001.5244). The patients/participants provided their written informed consent to participate in this study. Written informed consent was obtained from the individual(s) for the publication of any potentially identifiable images or data included in this article.

## Author Contributions

KR and MP designed the research studies. LS, LNM, LN, and LFM collected the material and clinical exams. KR, NS, PP, and LH conducted the experiments. KR acquired the data and wrote the manuscript. KR, EP, and MP analyzed the data. MP, JC, AT, RB-O, and FS provided the reagents. DP and MP contributed to reading the manuscript critically. All authors agreed with the manuscript.

## Conflict of Interest

The authors declare that the research was conducted in the absence of any commercial or financial relationships that could be construed as a potential conflict of interest.
