# Supplementary material for: Zika Induces Human Placental Damage and Inflammation
Source: Front Immunol. 2020 Sep 1;11:2146. doi: 10.3389/fimmu.2020.02146 (PMC7490298; doi:10.3389/fimmu.2020.02146)
Supplement: Supplementary file 1 [file Table_1.docx]

|  | **Histopathological changes** | **PRNT titer** |
| --- | --- | --- |
| **Case 1** | Acute and chronic deciduitis, chronic intervillositis, intervillous congestion, dysmorphic villi. | - |
| **Case 2** | Edema, hemorrhage, endothelial thickening, immature chorionic villi and chronic deciduitis, extramedullary hemopoiesis, perivascular fibrosis, villositis and stromal fibrosis. | 1/20 |
| **Case 3** | Immature chorionic villi, chronic and acute deciduitis, Hofbauer's cell hyperplasia, villositis and intervillositis | 1/20 |
| **Case 4** | Excessive syncytial nodes, intervillous congestion, fibrinoid necrosis, immature chorionic villi and basal focal chronic villositis. | 1/640 |
| **Case 5** | Chronic deciduitis, immature chorionic villi, Hofbauer's cell hyperplasia and intervillositis | - |
| **Case 6** | Endothelial thickening, fibrin areas, infarct, calcification, chronic deciduitis, basal chronic villositis and immature chorionic villi. | 1/80 |
| **Case7** | Focal acute and chronic deciduitis, excessive syncytial nodes, intervillous congestion, immature chorionic villi and basal focal chronic villositis. | - |
| **Case 8** | Fibrin areas, immature chorionic villi, villositis, acute deciduitis, ischemia from chronic maternal vascular hypoperfusion, villous rarefaction (secondary to ischemia) and hypoplasia. | 1/10 |
| **Case 9** | Endothelial thickening, ischemia from chronic maternal vascular hypoperfusion, vascular congestion, calcification, immature chorionic villi and extramedullary hemopoiesis. | 1/20 |
| **Case 10** | Endothelial thickening, calcification, acute and chronic deciduitis, basal chronic villositis and immature chorionic villi. | - |

**Supplementary Material**

**Supplementary Table 1. Description of all histopathological changes found in the patients' HE stained slides and PRNT titers.**

## Supplementary Figures

**
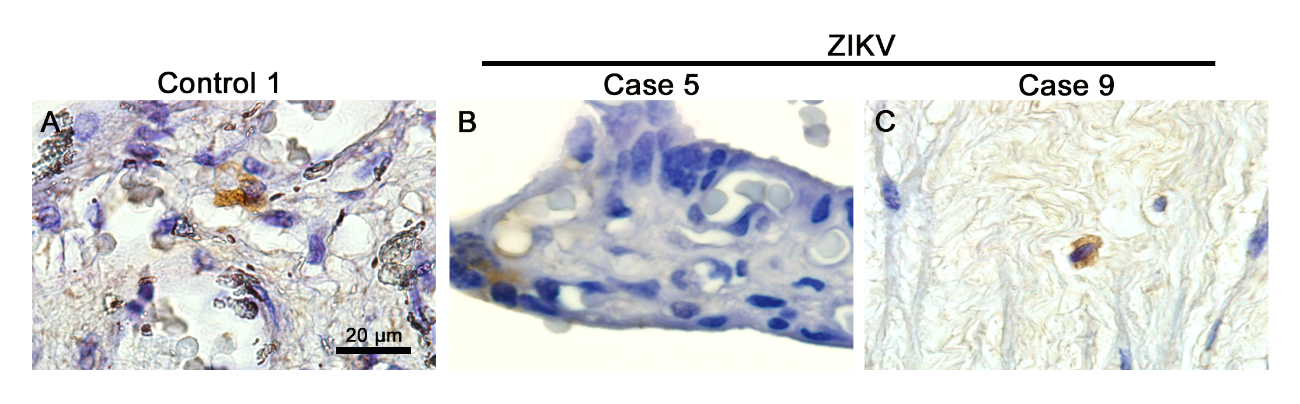
**

**Supplementary Fig. 1- Detection of T CD4+ Lymphocytes in control and ZIKV-infected placental tissues**. **A**) Detection of CD4+ cells by immunohistochemistry in chorionic villi of control placenta. **B-C**) Detection of CD4+ cells by in chorionic villi and decidua of infected placentae.
